# Influence of vacuum chamber impurities on the lifetime of organic light-emitting diodes

**DOI:** 10.1038/srep38482

**Published:** 2016-12-13

**Authors:** Hiroshi Fujimoto, Takashi Suekane, Katsuya Imanishi, Satoshi Yukiwaki, Hong Wei, Kaori Nagayoshi, Masayuki Yahiro, Chihaya Adachi

**Affiliations:** 1 Fukuoka i^3^-Center for Organic Photonics and Electronics Research (i^3^-OPERA), 5-14 Kyudai-shinmachi, Nishi, Fukuoka 819-0388, Japan; 2 Center for Organic Photonics and Electronics Research (OPERA), Kyushu University, 744 Motooka, Nishi, Fukuoka 819-0395, Japan; 3 Technology Innovation Center, Sumika Chemical Analysis Service, Ltd. (SCAS), 7-5, Kikumoto-cho 1-chome, Niihama, Ehime, 792-0801, Japan; 4 Technology Innovation Center, Sumika Chemical Analysis Service, Ltd. (SCAS), 1-135, Kasugade-Naka 3-chome, Konohana-ku, Osaka, 554-0022, Japan; 5 Osaka Laboratory, Sumika Chemical Analysis Service, Ltd. (SCAS), 1-135, Kasugade-Naka 3-chome, Konohana-ku, Osaka, 554-0022, Japan; 6 Institute of System, Information Technology and Nanotechnology (ISIT), 2-1-22 Momochihama, Sawara, Fukuoka 819-0395, Japan; 7 Japan Science and Technology Agency (JST), ERATO, Adachi Molecular Exciton Engineering Project, Fukuoka 819-0395, Japan; 8 International Institute for Carbon Neutral Energy Research (WPI-I^2^CNER), Kyushu University, 744 Motooka, Nishi, Fukuoka 819-0395, Japan

## Abstract

We evaluated the influence of impurities in the vacuum chamber used for the fabrication of organic light-emitting diodes on the lifetime of the fabricated devices and found a correlation between lifetime and the device fabrication time. The contact angle of the ITO substrates stored the chamber under vacuum were used to evaluate chamber cleanliness. Liquid chromatography-mass spectrometry was performed on Si wafers stored in the vacuum chamber before device fabrication to examine the impurities in the chamber. Surprisingly, despite the chamber and evaporation sources being at room temperature, a variety of materials were detected, including previously deposited materials and plasticizers from the vacuum chamber components. We show that the impurities, and not differences in water content, in the chamber were the source of lifetime variations even when the duration of exposure to impurities only varied before and after deposition of the emitter layer. These results suggest that the impurities floating in the vacuum chamber significantly impact lifetime values and reproducibility.

Advances in carbon-based semiconductors have organic electronics close to realizing a wide variety of lightweight, low-cost, flexible, and energy-efficient devices including light-emitting diodes, solar cells, and transistors that will have an enormous impact on our daily lives. Organic light-emitting diodes (OLEDs), the most advanced of these technologies, already form the basis for a new generation of commercially viable smart phone displays and large-screen televisions^1^ and are being developed for flexible lighting and display panels, which cannot be realized with inorganic LED or LCD technology^2^,^3^. Although progress has been rapid regarding many facets of the performance, processing, and development of organic electronics, difficulty obtaining reproducible operational lifetimes for organic devices fabricated with the same structure and materials, even when made in the same laboratory, is a problem that continues to mystify researchers. Here we show that small amounts of impurities floating in the chamber used to fabricate vacuum-deposited OLEDs are a source of large variations in operational lifetime even when the time spent in the chamber differs by only a short amount. Our results suggest that reproducible and long lifetimes can be obtained in OLEDs and other organic devices that include a vacuum-deposition step, such as solar cells and transistors, without the use of ultra-high vacuum chambers by controlling the device fabrication and cleanliness of the chamber. Furthermore, the strong influence of miniscule amounts of impurities on lifetime found here highlights a major challenge facing organic devices fabricated by spin-coating, inkjet, and other printing methods, for which elimination of impurities is more difficult.

Practical OLEDs rely on organic materials with high electroluminescence efficiencies and good stability under electrical operation. Internal quantum efficiencies (*η*_int_) of 100% have already been achieved through the development of second-generation emitter materials based on phosphorescence^4^ and, more recently, third-generation emitters based on thermally-activated delayed fluorescence (TADF), with TADF emitters eliminating the need for the rare metals used in phosphorescent materials^5^,^6^. A long lifetime is also critical for commercial OLEDs, so research to develop durable device structures, reduce driving current by improving light out-coupling efficiency^7^,^8^,^9^,^10^,^11^,^12^,^13^,^14^, and understand degradation mechanisms is in progress worldwide^15^,^16^,^17^,^18^,^19^,^20^,^21^,^22^,^23^,^24^,^25^,^26^,^27^,^28^,^29^.

The lifetime of OLEDs, which combine a large number of organic materials, must be evaluated with good reproducibility to advance this area, but experimentally measured lifetimes often vary depending on conditions related to things such as the vacuum chamber, location, date, and operator. The difference between lifetimes measured for devices fabricated in academic laboratories and for those being mass produced can be particularly large. However, very few studies discuss why lifetimes for OLEDs fabricated with the same structure and materials can vary so widely.

The degradation mechanisms affecting the lifetime of OLEDs can be either intrinsic or extrinsic. Degradation mechanisms intrinsic to the organic materials or device structure include interfacial degradation such as the organic/cathode or organic/anode interface^17^,^18^, trap formation^19^, and polaron-exciton interactions^20^. Chemical reactions and Joule heating have also been reported to cause bulk degradation^21^,^22^,^23^. Thus, researchers have been actively developing materials and device structures to prevent such intrinsic degradation.

Impurities on the indium tin oxide (ITO) substrate surface are one source of extrinsic degradation that can lower lifetime, and the characteristics and lifetime of OLEDs can be improved by exposure to UV or O_2_ plasma, which decompose organic impurities on the ITO surface^26^,^27^,^28^. Contamination from the outgassing of sealing resin^29^ and halogen impurities in the organic material^30^,^31^ are another two sources of degradation that have been identified. Furthermore, residual water in the vacuum chamber during fabrication^24^,^25^, which particularly affects the interface between the hole transport layer and the emission layer, has been shown to reduce lifetime by participating in degradation-inducing electrochemical reactions with the organic materials. However, the influence of impurities other that water in the deposition chamber is still poorly studied even though the possibility of such impurities being in the chamber is high.

Impurities in the vacuum chamber can arise from a variety of sources that are often overlooked. Although the development oil-free vacuum pumps eliminated oil pollution as a problem, many key components of the vacuum chamber still require vacuum grease, which can outgas when heated such as by the evaporation sources, and residual traces of the oil used during the processing the stainless steel parts of a chamber can also be released in vacuum^32^,^33^,^34^. Most chambers rely on some polymer-based parts such as O-rings and insulating resins, which have outgassing rates much higher than those of metal components, and such polymer materials can outgas unreacted components (*e.g.*, plasticizers and curing agents), decomposition products, and absorbed gases^35^. Even with the multitude of potential contamination sources in the vacuum chamber, the influence of such impurities on OLED lifetime has yet to be studied in detail.

To fill this gap and clarify the factors that affect lifetime reproducibility in OLEDs, we investigated the impurities in an OLED vacuum chamber and their impact on the lifetime of highly efficient TADF-based OLEDs. We find that the length of time the devices spend in the chamber during fabrication can greatly affect the lifetime even though similar initial characteristics are obtained for the devices. These results indicate that longer lifetimes in organic devices can be achieved by shortening the device fabrication time. To determine if impurities in the vacuum chamber could be the cause of the variations, we analyze the impurities adsorbed on substrates kept in the chamber for various durations using contact angle, liquid chromatography-mass spectrometry (LC-MS), and wafer thermal desorption gas chromatograph mass spectrometry (WTD-GC-MS) and find a correlation between the number of chamber impurities and the device lifetime.

## Results

### Relationship between lifetime and device fabrication time

**Figure 1:**
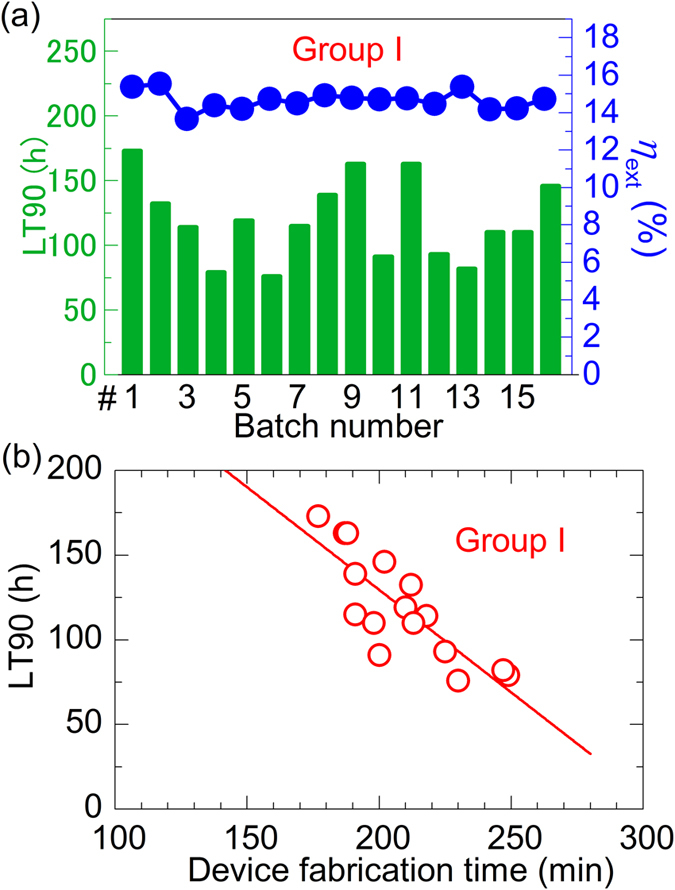
Initial characteristics and lifetime of Group I devices. (**a**) The *η*_ext_ and lifetime of the TADF devices made in separate batches numbered in chronological order. (**b**) Lifetime as a function of device fabrication time, which is the time from the beginning of the HAT-CN deposition until the end of the Al deposition. Values are all averaged for two to four devices per batch.

We first evaluated the batch-to-batch reproducibility of OLED characteristics and lifetime by measuring devices with the same structure fabricated in 16 separate batches over a span of four months (**Group I**). Figure 1(a) shows the external quantum efficiencies (*η*_ext_) and lifetimes of the TADF devices in the chronological order of their deposition. The *η*_ext_ were measured at 1000 cd/m^2^, and the lifetime (LT90) is the time it took for the luminance to drop to 90% of the initial (1000 cd/m^2^) when operated at a constant current. Table 1 summarizes the initial characteristics at 1000 cd/m^2^ and lifetime of these devices (**Group I**), and Supplementary Fig. S1 shows typical *J*-*V*-*η*_ext_ characteristics measured for the OLEDs.

Although the initial characteristics of the devices can be well reproduced from batch to batch, the lifetime showed a large variance with values ranging from 76 h to 173 h. Since the lifetimes in this case do not correlate with the *η*_ext_, the lifetime variation cannot be explained by a difference in the current density at 1000 cd/m^2^. On the other hand, a correlation between device fabrication time, defined here as the total time from the beginning of the HAT-CN deposition until the end of the Al deposition, and lifetime could be found, as shown in Fig. 1(b) with lifetime decreasing as the device fabrication time increases. Variations in the device fabrication time arise from the time needed to adjust the deposition rate of each layer before beginning to deposit the layer on the substrate. Therefore, the device fabrication time is a factor that must be considered and controlled when trying to compare lifetimes.

Because we think that organic impurities in the chamber may be affecting the lifetime, we surveyed the impurities that deposit on clean Si wafers kept in the vacuum chamber for different durations. The evaporation sources were kept at room temperature while the Si wafers were stored in the chamber. On the surface of a wafer stored in the chamber for 30 min, 13 materials were detected using LC-MS. Storing a Si wafer in the chamber for 15 h resulted in the detection of an additional 36 materials, for a total of 49 detected materials, and the ion counts increased for 12 of the 13 materials also detected for the 30-min wafer, indicating an increase in the deposited amount of those materials. Thus, the substrate can be contaminated just by being in the chamber, and the amount of these impurities mixed in the device will increase with the device fabrication time.

Furthermore, the contact angles measured on three occasions over the period of the experiments for ITO substrates that were loaded in the vacuum chamber for 30 min before loading the device substrates increased from 5° before loading to 19–25° after storage in the chamber. These results suggest that the organic impurities detected by LC-MS deposited in high enough amounts to raise the contact angle. In light of these device, LC-MS, and contact angle results, we suspected that the impurities in the vacuum chamber are an important factor contributing to batch-to-batch variations in lifetime.

### Dependence of lifetime on impurities and water in vacuum chamber

In addition to the organic impurities, water in the vacuum chamber is well known to decrease lifetime^24^,^25^ and could lead to a correlation between device fabrication time and lifetime. Therefore, we next tried to experimentally isolate the effects of the water and impurities in the vacuum chamber on the lifetime. The following experiments were all performed two months after the **Group I** experiments, and materials other than those used in the devices fabricated here were deposited in the chamber during this span.

Since we expect that many of the impurities measured by LC-MS are residue from previous depositions that are released from the vacuum chamber walls during device fabrication, we tried to remove those impurities by cleaning the chamber. Deposition shields were replaced with clean shields, and the surfaces in the chamber were wiped with acetone to remove deposited organic materials. After high-vacuum evacuation, the evaporation sources were heated to their maximum temperature to complete the cleaning. The amount of water was expected to be equal before and after cleaning while the amount of organic impurities should be reduced.

**Figure 2:**
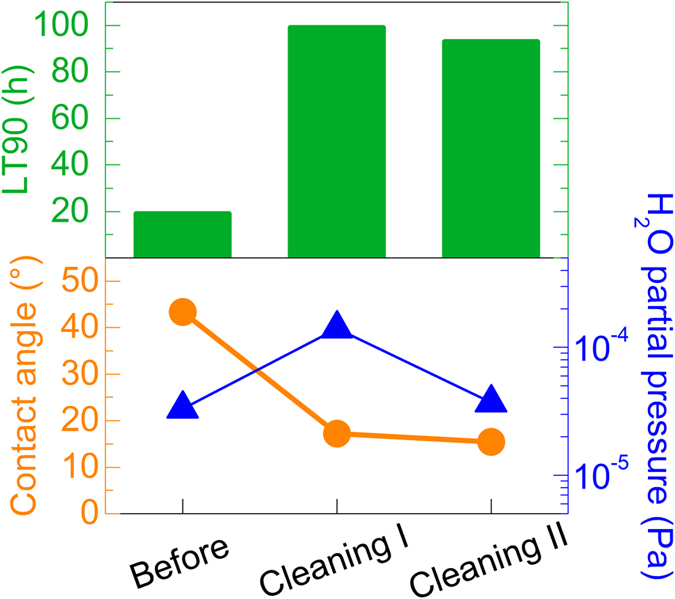
Effect of chamber cleaning on lifetime and vacuum environment. Device lifetime (bars), contact angles of ITO substrate stored in the vacuum chamber for 30 min before deposition (circles), and partial pressures of water during deposition for the batches fabricated before cleaning (**Before**), after chamber cleaning and evacuation to high vacuum overnight (**Cleaning I**), and evacuated to high vacuum for an additional two days (**Cleaning II**).

Figure 2 shows the device lifetime and the partial pressure of water for batches of devices fabricated before cleaning (**Before**), after chamber cleaning and high-vacuum evacuation overnight (**Cleaning I**), and after high-vacuum evacuation for an additional 2 days following the deposition of **Cleaning I** (**Cleaning II**). To eliminate its influence, the device fabrication time of all devices was 160 min. Although the partial pressure of water measured by Q-mass during deposition increased from 3.3 × 10^−5^ Pa for **Before** to 1.4 × 10^−4^ Pa for **Cleaning I**, the device lifetime after cleaning greatly improved from 19 h to 99 h. On the other hand, the lifetimes were nearly the same for **Cleaning I** and **Cleaning II** even though the partial pressure of water during **Cleaning II** (3.7 × 10^−5^ Pa) was lower. Thus, the increased amount of water during **Cleaning I** appears to have had little effect on the lifetime. Although Yamamoto *et al*. demonstrated a great decrease in lifetime when devices were fabricated in the ultra-high vacuum region with a water partial pressure of 3 × 10^−7^ Pa and water amounts ranging over four orders of magnitude were introduced by storing the incomplete devices in a chamber with a water partial pressure of 3 × 10^−4^ Pa for varying lengths of times^25^, the negligible influence in this paper is reasonable since the increase in water was much smaller (only a four-fold increase in partial pressure).

The impurities were again examined using LC-MS on Si wafers that were stored in the vacuum chamber for 15 h before fabricating either the **Before** or **Cleaning I** batches of devices. Although 88 materials were detected for both batches, the quantity of 58 of the materials decreased after chamber cleaning, leading to a decrease in the total ion count for all detected impurities of more than 15%. The contact angle of ITO substrates stored in the chamber for 30 min before loading the device substrates improved from 43° after cleaning to 17° after cleaning. Although the detected organic impurities after cleaning were still sufficient to affect the contact angle, the increase in contact angle was greatly reduced by cleaning. Thus, the impurities in the vacuum chamber were correlated with the lifetime, and changes in the amount of water in the chamber at the levels measured here had little influence.

### Analysis of impurities in vacuum chamber

**Figure 3:**
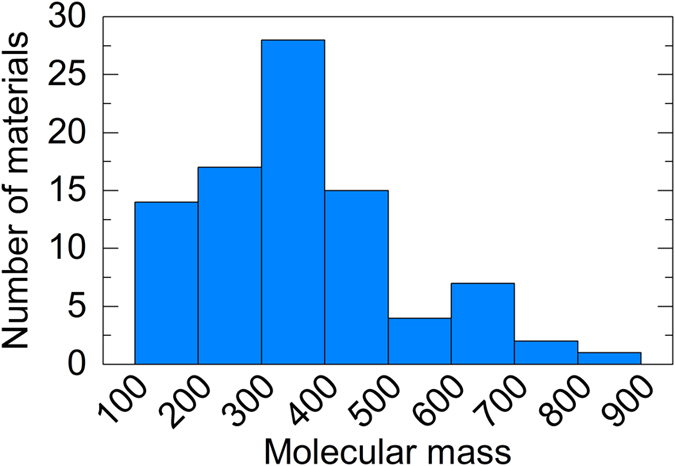
Molecular masses of impurities. Histogram showing the distribution of molecular masses of the detected materials.

We further analyzed the impurities in the chamber to better understand their origin. Figure 3 shows a histogram separating the materials detected in the previous section by molecular mass. Interestingly, not only low-molecular-mass materials but also high-molecular-mass materials were detected in the vacuum chamber. We can tentatively assign some of the signals to α-NPD (**1**), Tris-PCz (**2**), TPBi (3), T2T (**4**), TCTA (**5**), C_13_H_10_N_2_ (**6**), bis(2-ethylhexyl) adipate (DOA, **7**), C_18_H_34_O_4_ (**8**), diisononyl phthalate (**9**), and C_22_H_42_O_5_ (**10**) (see Supplementary Fig. S2 for the chemical structures) based on the exact molecular masses and compositions calculated by LC-MS analysis. Surprisingly, despite the chamber and evaporation sources being at room temperature, a variety of previously deposited materials (**1**–**5**) and their fragments or degradation products (**6** likely originates from TPBi) were detected. Furthermore, compounds that most likely do not originate from the materials used for OLED active layers (**7**–**10**) were detected. These include DOA, which is used in many chambers as a vacuum grease, and diisononyl phthalate, which is a well-known plasticizer for imparting low-temperature flexibility to polyvinyl chloride or rubber. These materials could be released from the resins used for insulation, the grease coating vacuum components, and the O-rings. Using LC-MS analysis, similar impurities floating in a different evaporation chamber having a different design and being operated under different conditions were also found (see Supplementary Note 2).

**Figure 4:**
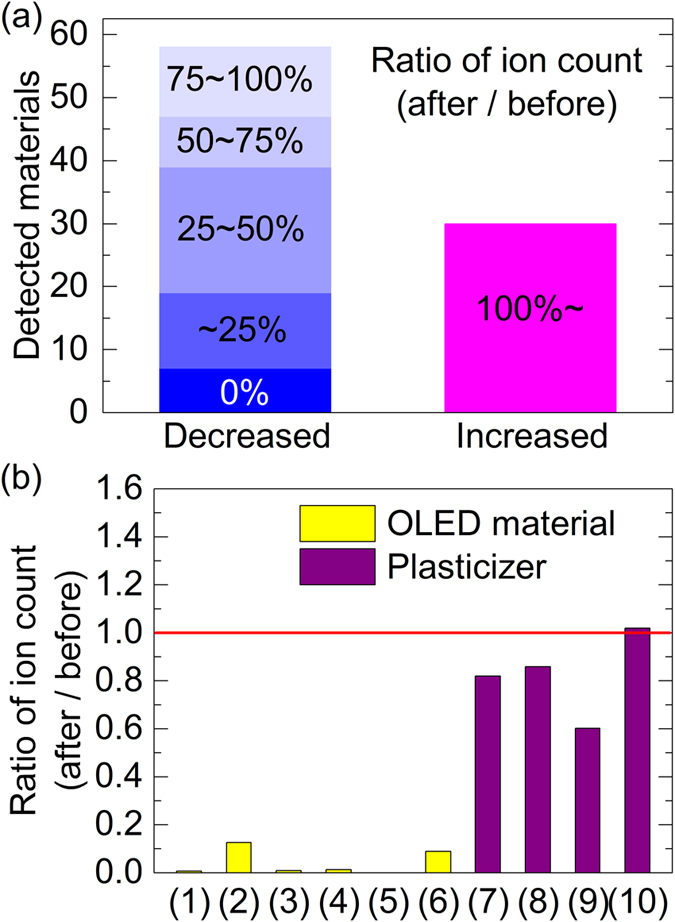
Analysis of LC-MS data after chamber cleaning. (**a**) Breakdown of the ion counts after cleaning as a percentage of those before cleaning for the detected materials. (**b**) Ratios of the ion counts before cleaning to those after cleaning for the identified impurities (see Supplementary Fig. S2).

Figure 4(a) groups the detected materials by their amount after cleaning as a percentage of that before cleaning. Of the 58 materials that decreased in quantity by chamber cleaning, about two thirds decreased by more than 50%, and the majority of the materials that decreased by more than 75% had molecular masses greater than 500 (Supplementary Fig. S3). The breakdown in Fig. 4(b) of the changes in ion counts based on the identified signals shows that the signals from the OLED materials, **1**–**5**, and their fragments or degradation products, **6**, were significantly reduced by chamber cleaning (yellow bars). This indicates that such materials can be removed by wiping the chamber and replacing the deposition shields with clean ones. In contrast, the signals proposed to originate from plasticizers, **7**–**10**, decreased much less (only 15–40%) and actually increased in one case after chamber cleaning (purple bars). Since plasticizers are incorporated in the chamber components, the source of these materials cannot be greatly reduced simply by cleaning.

Thus, cleaning the chamber in this manner can actually lead to a short-term increase in the release of some impurities. One possibility is that debris released from crevices and corners in the chamber by mechanical scraping may not be completely removed by wiping with solvent and instead leave a thin film of residue that can be more easily discharged in the chamber under vacuum. Decreasing the amount of these impurities by rethinking the methods for chamber cleaning and the materials used in chamber components could help to improve lifetime.

### Device fabrication time and amount of impurities

Having established that deposition time affects lifetime because of impurities in the chamber, we more deeply investigated the correlation between lifetime and device fabrication time by increasing or decreasing the waiting time equally before and after the emission layer (EML) deposition while keeping the deposition timing of all other layers constant (**Group II**). Other parameters such as the deposition rates (see Methods for the detailed rates) for the different layers were controlled to be nearly the same for each batch, and only the waiting time was varied. In addition, the organic materials and evaporation source surroundings were degassed by evaporating amounts of materials corresponding to at least the same thicknesses as used in the devices after any exposure of the organic chamber to atmosphere such as to load more organic material. This degassing procedure resulted in a vacuum pressure that was relatively constant during the deposition of the organic layers.

To analyze the change in impurities, Si wafers for LC-MS analysis were loaded with the device substrates during the device fabrication. The Si wafers were covered with non-contact shadow masks during deposition but exposed to the vacuum the same as the device substrates at all other times. Since these experiments were performed in three series over a span of three months, the chamber conditions were different. Even so, the contact angles were within the range of 10–18° after chamber cleaning before each series of experiments, and the initial device characteristics were reproducible (**Group II** in Table 1). Thus, regardless of the time, the contamination was assumed to be relatively constant.

**Figure 5:**
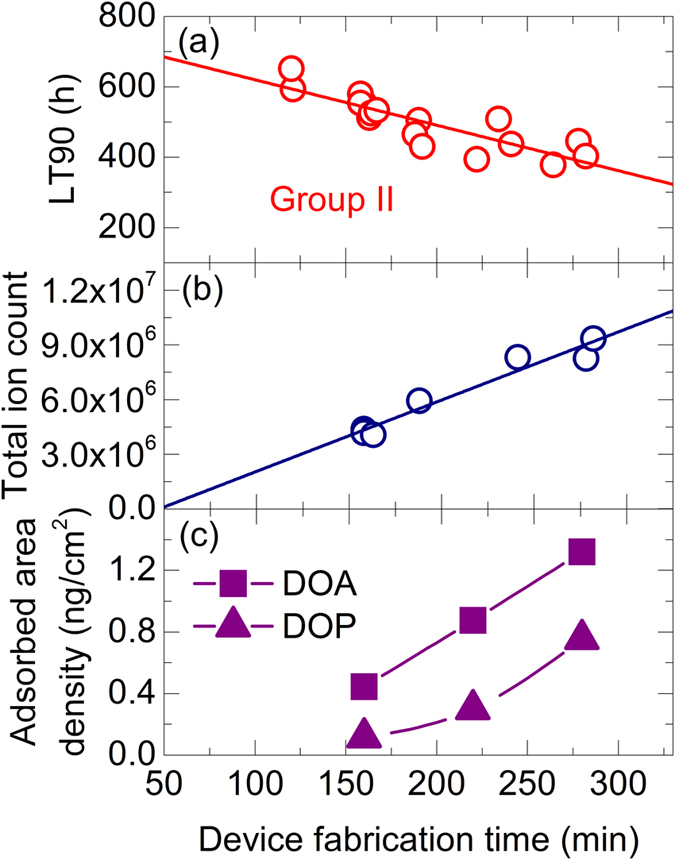
Effect of fabrication time on lifetime and vacuum environment. (**a**) Dependence of the lifetime on the device fabrication time for the **Group II** devices when the waiting times before and after EML deposition were equally extended or shortened. Lifetime values of 12 of the batches were averaged for two to four devices each, while only a single lifetime was available for the other five batches. (**b**) Total ion count for impurities measured by LC-MS on Si wafers loaded with the devices during fabrication. (**c**) Impurity quantity measured by WTD-GC-MS for DOA (squares) and DOP (triangles).

Figure 5(a) shows the dependence of the lifetime on the device fabrication time, which is the time from the start of the HAT-CN deposition to the end of the Al deposition, for a variable EML waiting time. The lifetime and the device fabrication time were correlated even when only the time before and after the EML deposition was changed. A clear correlation is also found between the device fabrication time and the total ion count of impurities on the Si wafers plotted in Fig. 5(b), the *x*-intercept of which corresponds to the ~40 min of deposition time when the Si wafers were masked. Further, the contact angle of the ITO substrates stored in the chamber increased with storage time (Supplementary Fig. S4). These results suggest that the lifetime is impacted by impurities adsorbed at the EML interfaces since the effect of water was found to be small in our experiments.

Reproducible correlations between deposition amount and device fabrication time measured were found for some, but not all, of the impurities. Of the identified impurities, materials **7**, **8**, and **10** were found to deposit in amounts that reproducibly correlate with the device fabrication time when analyzed for deposition series performed one month apart (Supplementary Fig. S5). Among the unidentified impurities, 21 materials showed a correlation between their quantity and the lifetime, and 16 of these materials contained at least one oxygen atom, with some containing up to five oxygen atoms such as C_22_H_40_O_5_ or C_22_H_42_O_5_. Such oxygen-rich materials are expected to originate from the chamber components rather than the OLED source materials. In addition, materials containing nitrogen, phosphorus (C_11_H_17_OP), and chlorine (C_10_H_20_ONCl) were detected. Halogens such as chlorine are particularly known to influence the lifetime^30^,^31^. Thus, the correlations found here suggest that these materials are affecting the lifetime.

Considering the actually amount of some of the impurities from chamber components that can enter the device, the area density of DOA, a well-known plasticizer found in chamber components, absorbed on the Si wafers was confirmed to be on the sub-ng/cm^2^ order using WTD-GC-MS analysis (Fig. 5(c)). The amount of adsorbed bis(2-ethylhexyl)phthalate (DOP) was also detected on the sub-ng/cm^2^ order (Fig. 5(c)) with a strong correlation on the device fabrication time, and rough calculations suggest that these amounts could realistically cover enough of the surface to have an effect on the substrates and devices (see Supplementary Note 3). Thus, even extremely small amounts of the impurities mixing with the active layers during device fabrication can have a great impact on the device lifetime.

We also found that the dependence of lifetime on device fabrication time becomes weaker for lifetimes corresponding to a larger decrease in initial luminance (Supplementary Note 4). This suggests that extrinsic degradation caused by the impurities has a greater impact on the initial degradation than at later stages, which was also found for degradation from water in the chamber^36^. Mixed water in the organic film can be decomposed into H^+^ and OH^−^ ions by an electric field of a few MV/cm during device driving. Since these ions are very active, the organic materials in the device are decomposed, generating trap sites and quenchers^24^,^25^,^37^,^38^. The mixed impurities in the organic thin films found here might have similar effects. Further, by inserting an organic layer of just 1 nm in the EML interface, the charge trap density at the interface has been reported to greatly change^39^, suggesting that charge trapping could also be influenced by the extremely small amounts of impurities. Such reactions should increase with the amount of impurities and cease once the impurities have reacted, leading to a larger influence on the initial degradation.

## Discussion

We found that a major cause of batch-to-batch variation in the lifetime of OLEDs is differences in the device fabrication times. Although the sensitivity of device lifetime on fabrication time may vary for different chamber designs, these results indicate that device fabrication time should be controlled when making devices for lifetime comparison studies. Furthermore, our results suggest that extremely small amounts of impurities at the EML interfaces can greatly influence lifetime. Surprisingly, previously deposited OLED materials were found to be floating in the vacuum chamber even when the chamber and evaporation sources were at room temperature. We showed that wiping the vacuum chamber with solvent to clean it was particularly effective for reducing the impurities from previously deposited OLED materials and improved the lifetime.

Longer lifetimes might be achieved by further optimizing the cleaning method, and impurities should be considered during the design stage of vacuum equipment because of the effect of impurities from vacuum components on the device lifetime. For example, the use of resin materials that can release plasticizers into the vacuum chamber, thereby influencing lifetime, should be minimized. The processing of the stainless-steel chamber components during chamber fabrication should be reviewed because residual layers could form on the surface of the stainless steel after fabrication^40^. In particular, contaminants such as oil that diffused into the stainless-steel surfaces could be released when the chamber is under vacuum for extended periods. Electrolytic polishing can reduce surface roughness to achieve lower outgassing^32^,^41^, but phosphoric acid may remain after the polishing. Aluminum alloy can be a source of outgassing because a natural oxide layer with a thickness of 10 nm or more is formed on the surface of the alloy after machining. This porous oxide layer can readily adsorb impurities and gasses that can be released in vacuum^42^. These results shed new light on some of the factors affecting the reproducibility of OLED lifetime, which is one of the great mysteries of OLEDs. Ultimately, because of difficulty eliminating all contamination related issues, the most practical method for fabricating OLEDs is with the shortest process time possible.

## Methods

### Device fabrication and characterization

Devices were fabricated by thermal evaporation on to ITO-coated glass substrates pre-patterned with polyimide bank structures to define a circular active device area of 0.04 cm^2^ (Atsugi Micro Co., Ltd.). The substrates were cleaned in multiple solvent baths (see Supplementary Note 1 for more details) and stored in an oven at 80 °C until use. Before deposition of the active layers, the substrates were heated on a hot plate in an N_2_-filled glovebox at 250 °C for 30 minutes, treated with UV in a system connect to the glovebox, and then directly loaded into the load lock chamber of the deposition system. Active layer deposition began 15 min after loading into the deposition chamber.

The materials used in this experiment include 1,4,5,8,9,11-hexaazatriphenylenehexacarbonitrile (HAT-CN) as hole-injection layer (HIL), 9,9’,9”-triphenyl-9H,9’H,9”H-3,3’:6’,3”-tercarbazole (Tris-PCz) as hole-transport layer (HTL), 3,3-di(9H-carbazol-9-yl)biphenyl (mCBP) doped with (4s,6s)-2,4,5,6-tetra(9H-carbazol-9-yl)isophthalonitrile (4CzIPN) as emission layer (EML), 2,4,6-tris(biphenyl-3-yl)k,3,5-triazine (T2T) as hole-blocking layer, 2,7-bis(2,2’-bipyridine-5-yl)triphenylene (Bpy-TP2) as electron-transport layer, and LiF as electron-injection layer. Cathodes were deposited by evaporation of Al. The organic materials for each group of experiments were from the same synthetic lots to eliminate the influence of starting material purity. The material purities of Tris-PCz, mCBP, 4CzIPN, and T2T were 99.2%, 99.9%, 99.5%, and 99.4%, respectively, for Group I and 99.9%, 99.9%, 99.6%, and 99.9%, respectively, for Group II as evaluated by LC-MS. The results of elemental analysis for HAT-CN (calculated H: 0%, C: 56.26%, and N: 43.74%) were H: 0.07%, C: 56.44%, and N: 43.52% (same lot used for both groups). For Bpy-TP2 (calculated H: 4.51%, C: 85.05%, and N: 10.44%), elemental analysis found H: 4.47%, C: 85.15%, and N: 10.45% for Group I and H: 4.36%, C: 85.15%, and N: 10.46% for Group II. The device structure was ITO/HAT-CN (10 nm)/Tris-PCz (30 nm)/15% 4CzIPN:mCBP (30 nm)/T2T (10 nm)/Bpy-TP2 (40 nm)/LiF (0.8 nm)/Al (100 nm). The target of deposition rates of HAT-CN, Tris-PCz, T2T, Bpy-TP2, and LiF were 0.15 ± 0.05 Å/s, 0.5 ± 0.1 Å/s, 0.2 ± 0.05 Å/s, 0.5 ± 0.1 Å/s, and 0.04 ± 0.01 Å/s, respectively. The rate for Al deposition was 1 Å/s for the first 10 nm and then was increased up to 5 Å/s for the remainder. The EML was deposited once a stable deposition rate above 0.6 Å/s was obtained for mCBP to conserve material, so the deposition rate of CBP varied between 0.6 and 1 Å/s with 4CzIPN deposited at the corresponding rate for 15 wt%.

An external quantum efficiency measurement system (C9920-12, Hamamatsu Photonics K. K.) was used to measure the current-density-voltage characteristics and external quantum efficiency of the OLEDs. Device lifetime under constant-current driving conditions was measured at a temperature of 30 °C for an initial luminance of 1000 cd/m^2^ or an initial current density of 10 mA/cm^2^ using a lifetime measurement system (System Giken Co., Ltd.). The partial pressure of water in the vacuum chamber was measured using the quadrupole mass spectrometer (Q-mass) of a residual gas analyzer (Qulee HGM-302, ULVAC Inc.).

### Analysis of impurities in vacuum chamber

To examine the impurities, Si wafers were analyzed by LC-MS and WTD-GC-MS after storage in the vacuum chamber. Since obtaining the standard curves necessary to evaluate the absolute amounts of impurities detected by LC-MS^43^ would be impractical for the nearly 100 impurities that were found, we focus on the ion counts, which can indicate the relative change in impurity amount when compared for an individual materials. The LC-MS analyses were performed using a liquid chromatography system (LC-30AD, Shimadzu) equipped with a UV/Visible detector (SPD-20A, Shimadzu) and a mass detector (Exactive™, Thermo Scientific). The masses of adsorbed impurities were measured using WTD-GC-MS. The WTD-GC-MS analyses were performed using a silicon wafer analyzer (SWA-256, GL Sciences) in combination with a gas chromatography system (7890A GC, Agilent Technologies) and a mass spectrometer (5975C inert XL MSD, Agilent Technologies).

Contact angle measurements of ITO substrates stored in the chamber for 30 min before loading the device substrates were also performed to provide further evidence of the effect of impurities on surfaces in the chamber. The substrates were treated with a UV-O_3_ system (UV253, Filgen, Inc.), loaded in the vacuum chamber, and stored there for 30 minutes at a pressure in the 10^−6^ Pa range with the evaporation sources at room temperature. Contact angles were measured immediately after unloading the ITO substrates from the chamber using a DropMaster DMe-201 (Kyowa Interface Science Co., Ltd.) and water as the solution. The contact angles of ITO substrates immediately after UV-O_3_ treatment were less than 5°.

## Additional Information

**How to cite this article**: Fujimoto, H. *et al*. Influence of vacuum chamber impurities on the lifetime of organic light-emitting diodes. *Sci. Rep.* **6**, 38482; doi: 10.1038/srep38482 (2016).

**Publisher's note:** Springer Nature remains neutral with regard to jurisdictional claims in published maps and institutional affiliations.

## Supplementary Material

Supplementary Information

## Figures and Tables

**Table 1:** Initial characteristics and lifetime of the devices.

	Voltage^a^(V)	Current density^a^ (mA/cm^2^)	*η*_*ext*_^a^ (%)	Peak wavelength^a^ (nm)	Contact angle (°)	LT90^b^ (h)
Group I	5.10 ± 0.17	2.05 ± 0.13	14.7 ± 0.9	524.9 ± 2.2	19–25	76–173
Group II	5.02 ± 0.14	1.86 ± 0.08	16.1 ± 0.6	523.6 ± 2.2	10–18	378–652

^a^1000 cd/m^2^.

^b^Lifetime: Time until drop of luminance to 90% of the initial of 1000 cd/m^2^.
