# Supplementary material for: Influence of vacuum chamber impurities on the lifetime of organic light-emitting diodes
Source: Sci Rep. 2016 Dec 13;6:38482. doi: 10.1038/srep38482 (PMC5153845; doi:10.1038/srep38482)
Supplement: Supplementary Information [file srep38482-s1.doc]

**Supplementary Information**

**Influence of vacuum chamber impurities on the lifetime of organic light-emitting diodes**

Hiroshi Fujimoto^1,2 a)^, Takashi Suekane^3^, Katsuya Imanishi^4^, Satoshi Yukiwaki^1^, Hong Wei^5^, Kaori Nagayoshi^1^, Masayuki Yahiro^1,2,6^, and Chihaya Adachi^1,2,6,7,8 b)^

^1^Fukuoka i^3^-Center for Organic Photonics and Electronics Research (i^3^-OPERA), 5-14 Kyudai-shinmachi, Nishi, Fukuoka 819-0388, Japan

^2^Center for Organic Photonics and Electronics Research (OPERA), Kyushu University, 744 Motooka, Nishi, Fukuoka 819-0395, Japan

^3^Technology Innovation Center, Sumika Chemical Analysis Service, Ltd. (SCAS), 7-5, Kikumoto-cho 1-chome, Niihama, Ehime, 792-0801, Japan

^4^Technology Innovation Center, Sumika Chemical Analysis Service, Ltd. (SCAS), 1-135, Kasugade-Naka 3-chome, Konohana-ku, Osaka, 554-0022, Japan

^5^Osaka Laboratory, Sumika Chemical Analysis Service, Ltd. (SCAS), 1-135, Kasugade-Naka 3-chome, Konohana-ku, Osaka, 554-0022, Japan

^6^Institute of System, Information Technology and Nanotechnology (ISIT), 2-1-22 Momochihama, Sawara, Fukuoka 819-0395, Japan

^7^Japan Science and Technology Agency (JST), ERATO, Adachi Molecular Exciton Engineering Project, Fukuoka 819-0395, Japan

^8^International Institute for Carbon Neutral Energy Research (WPI-I^2^CNER), Kyushu University, 744 Motooka, Nishi, Fukuoka 819-0395, Japan

Electronic mail: ^a)^fujimoto@opera.kyushu-u.ac.jp, ^b)^adachi@cstf.kyushu-u.ac.jp


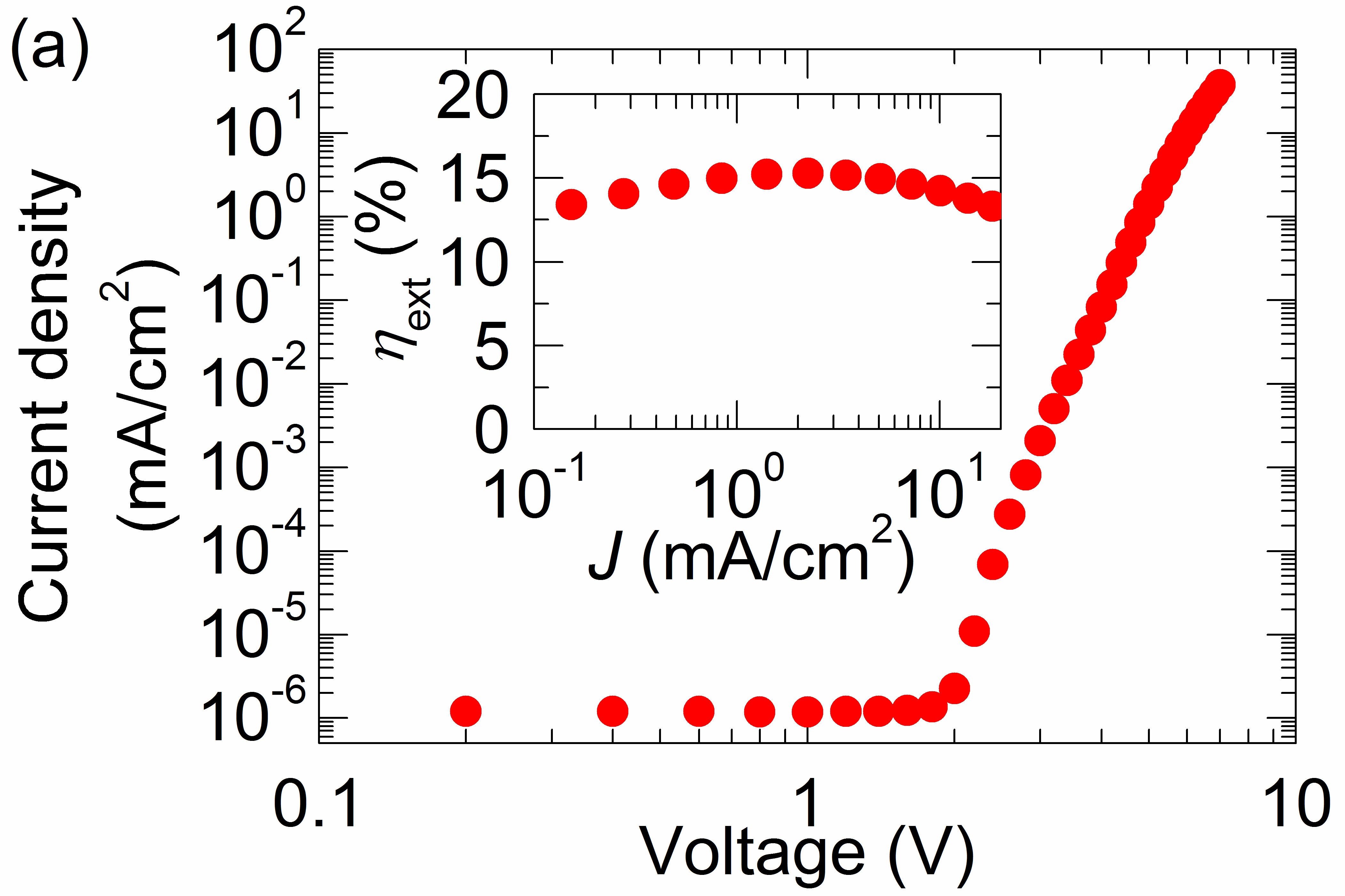


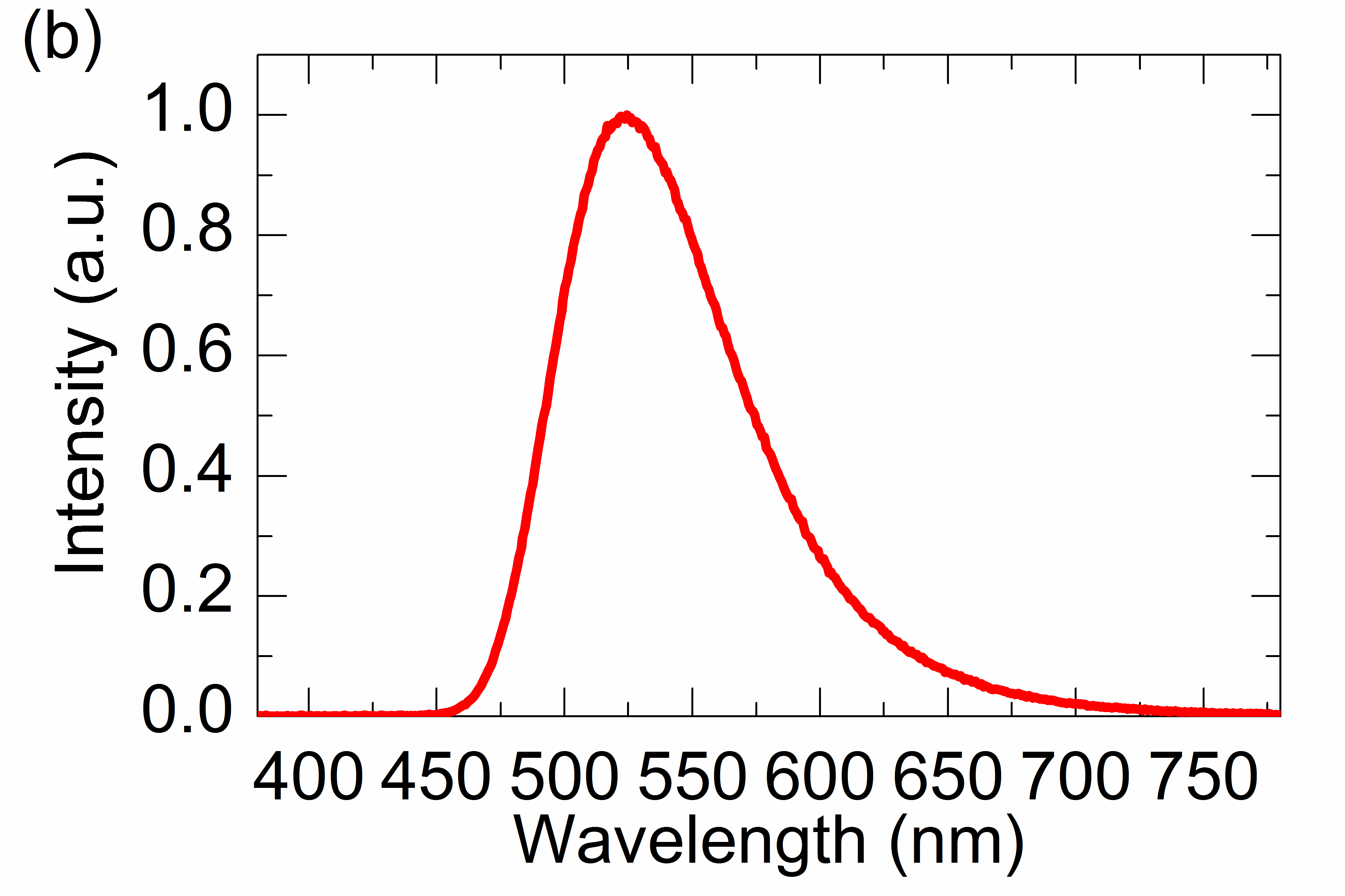


**Supplementary Figure S1. Electrical characteristics of a representative device.** (a) Representative *J*-*V* and (inset) *η*_ext_-*J* characteristics, and (b) emission spectra at *J* = 1 mA/cm^2^ of devices with the structure ITO (100 nm)/ HAT-CN (10 nm)/ Tris-PCz (30 nm)/ 15% 4CzIPN:mCBP (30 nm)/ T2T (10 nm)/ Bpy-TP2 (40 nm)/ LiF (0.8 nm)/ Al (100 nm).





**Supplementary Figure S2. Structures of some of the materials that could be tentatively assigned to LC-MS signals.** These materials provide representative groups of deposited OLED materials (**1**-**5**) and their fragment (**6** from TPBi) and materials thought to be used in chamber components (**7**-**10**).


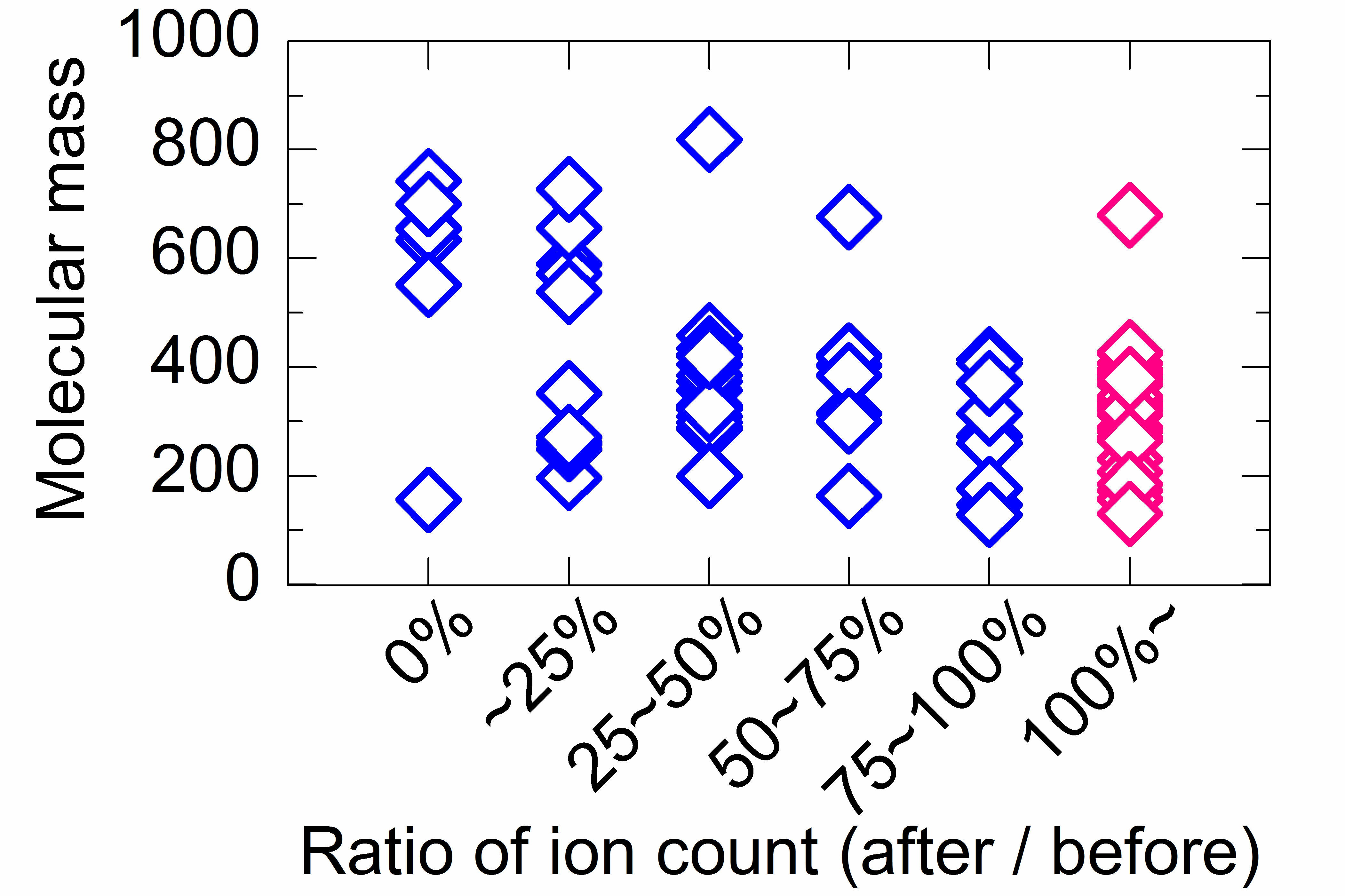


**Supplementary Figure S3. Effect of chamber cleaning on ion counts.** Relationship between the increase and decrease of materials after chamber cleaning and the molecular mass of the materials.


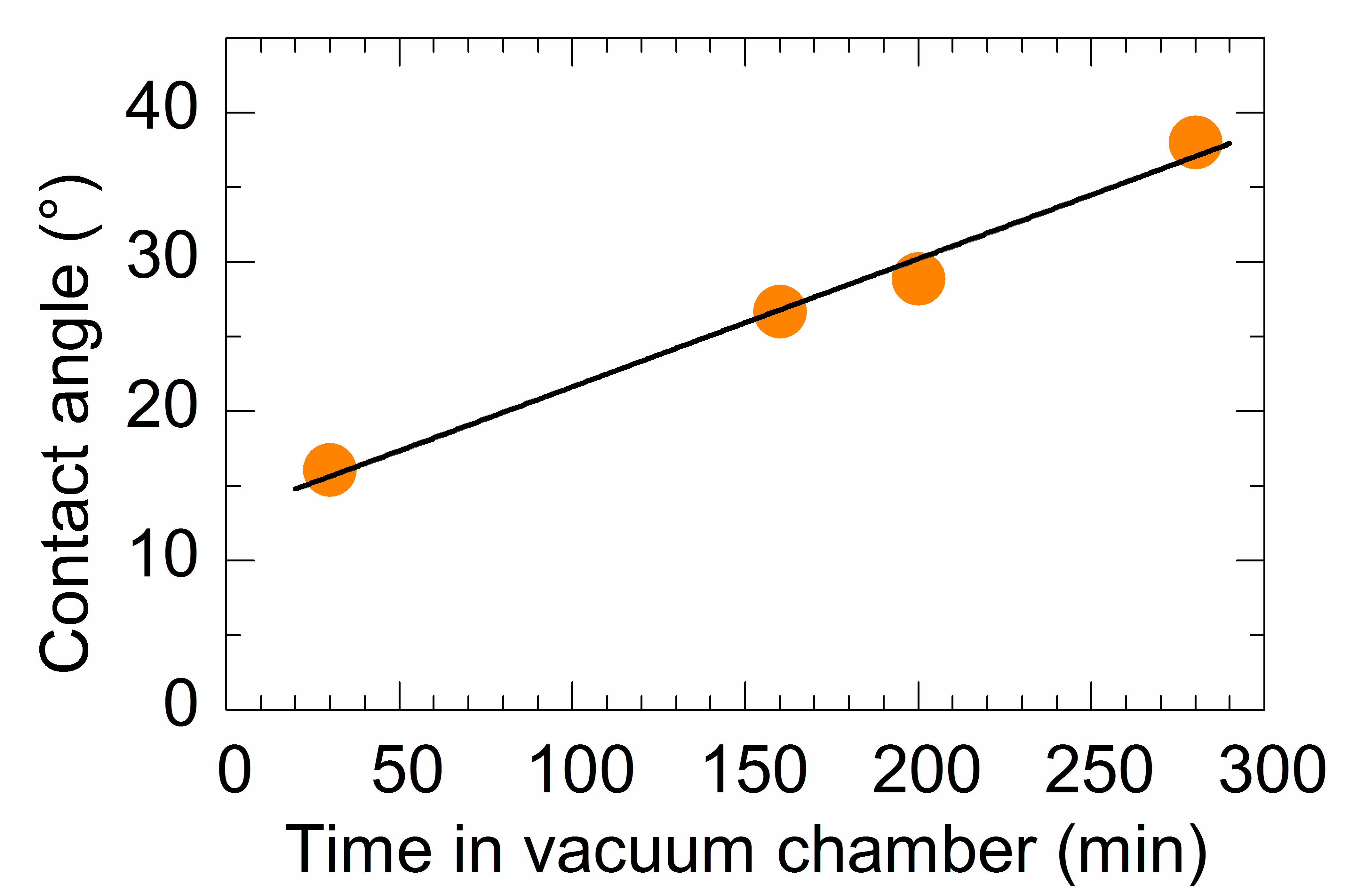


**Supplementary Figure S4. Effect of chamber environment on ITO substrates.** Dependence of the contact angle on the time an ITO substrate was stored in the vacuum chamber during the **Group II** experiments.


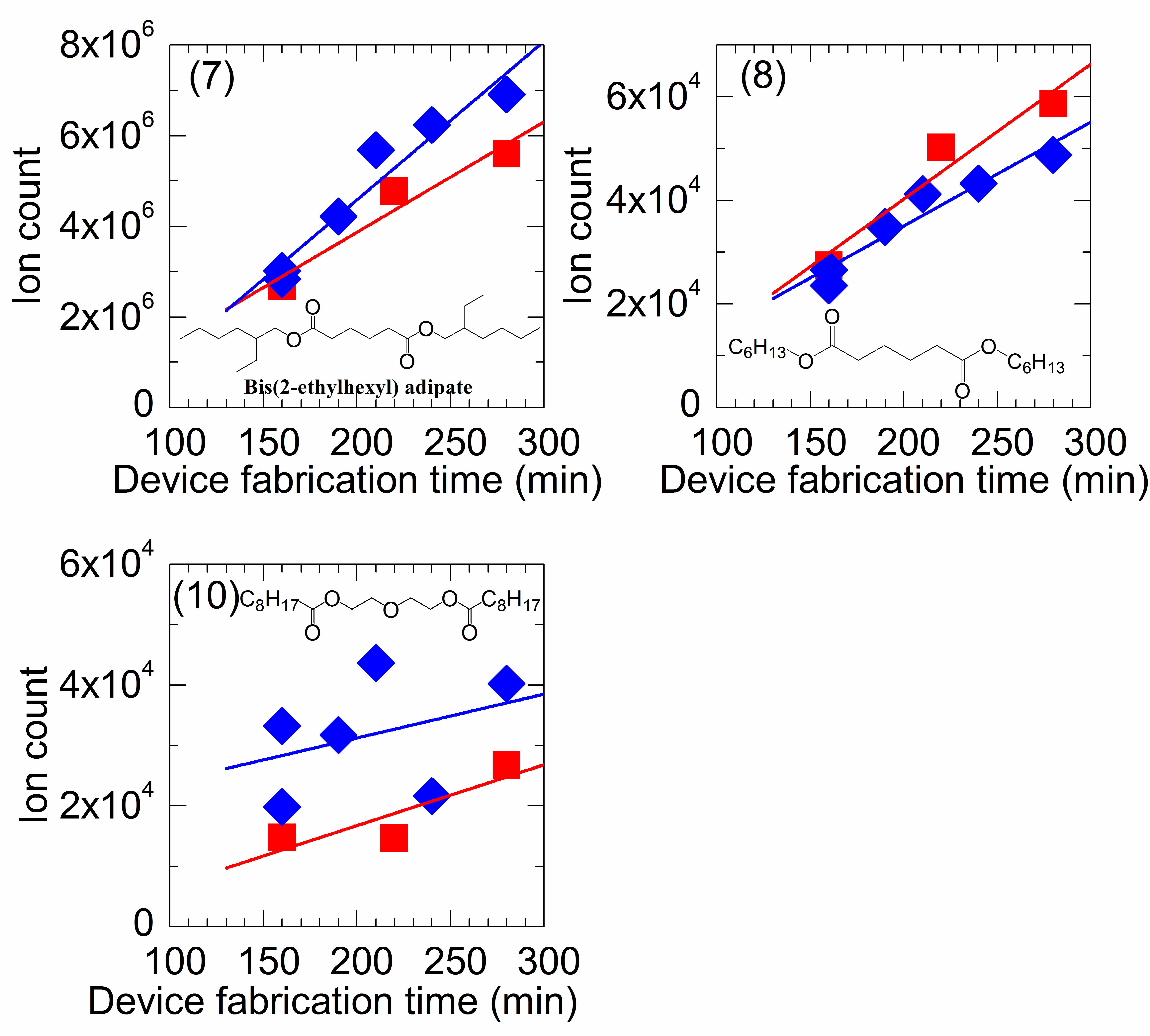


**Supplementary Figure S5. Dependence of ion count on fabrication time for select impuritires.** Dependence of the ion counts measured by LC-MS for some of the identified materials in Supplementary Fig. S2 on the device fabrication time. The measurements in red (squares) were performed about one month before those in blue (diamonds) during the **Group II** experiments. Materials **1**-**6** and **9** are not shown because they were detected in amounts too low for meaningful analysis.


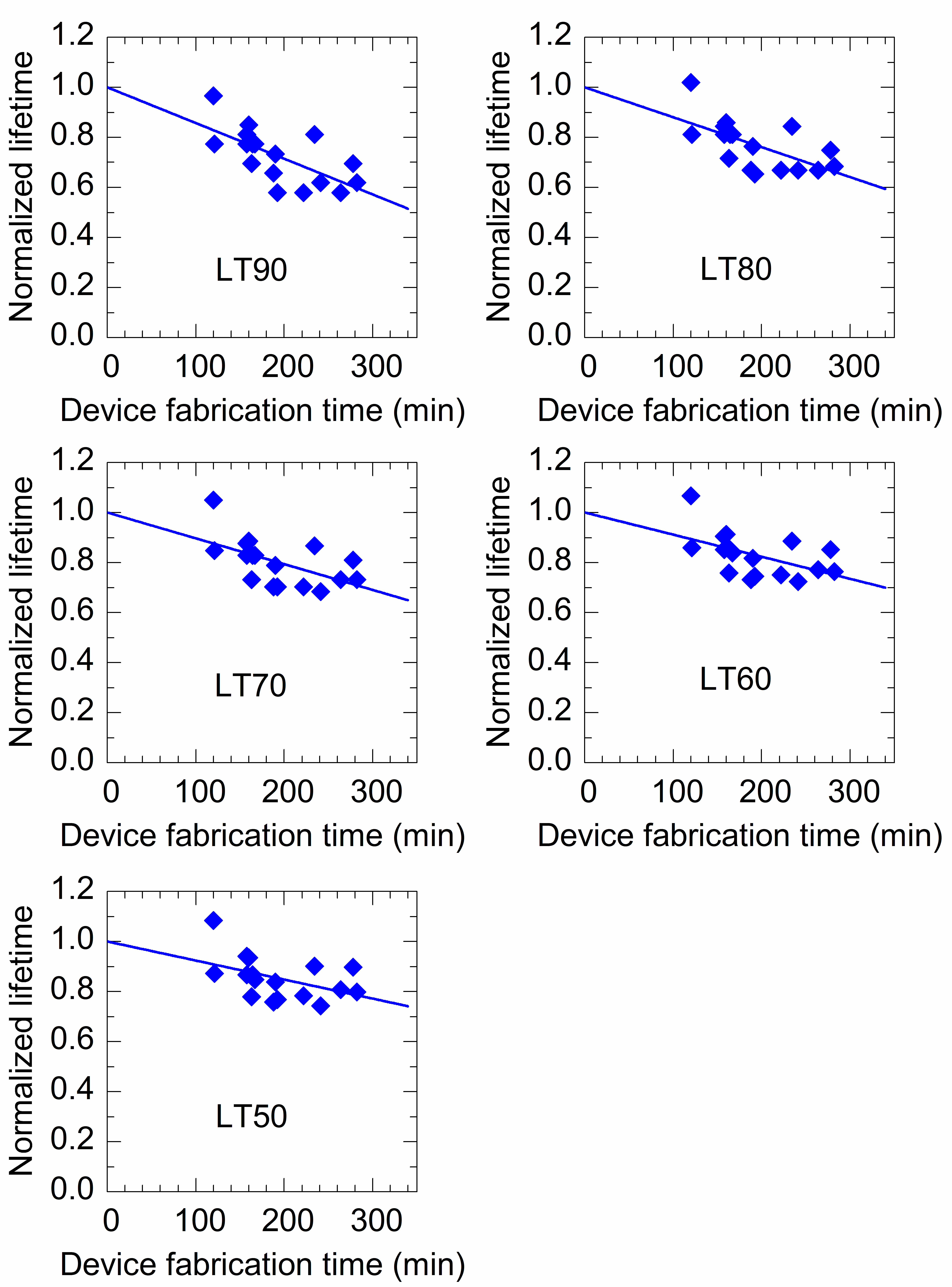


**Supplementary Figure S6 | Relationships for different lifetime thresholds.** Dependence of lifetime of the **Group II** devices on device fabrication time for different LT*x*, where *x* corresponds to final luminance as a percentage of the initial luminance. The values are normalized to the value of the *y*-intercepts, and the lifetimes were measured at a constant current of 10 mA/cm^2^.


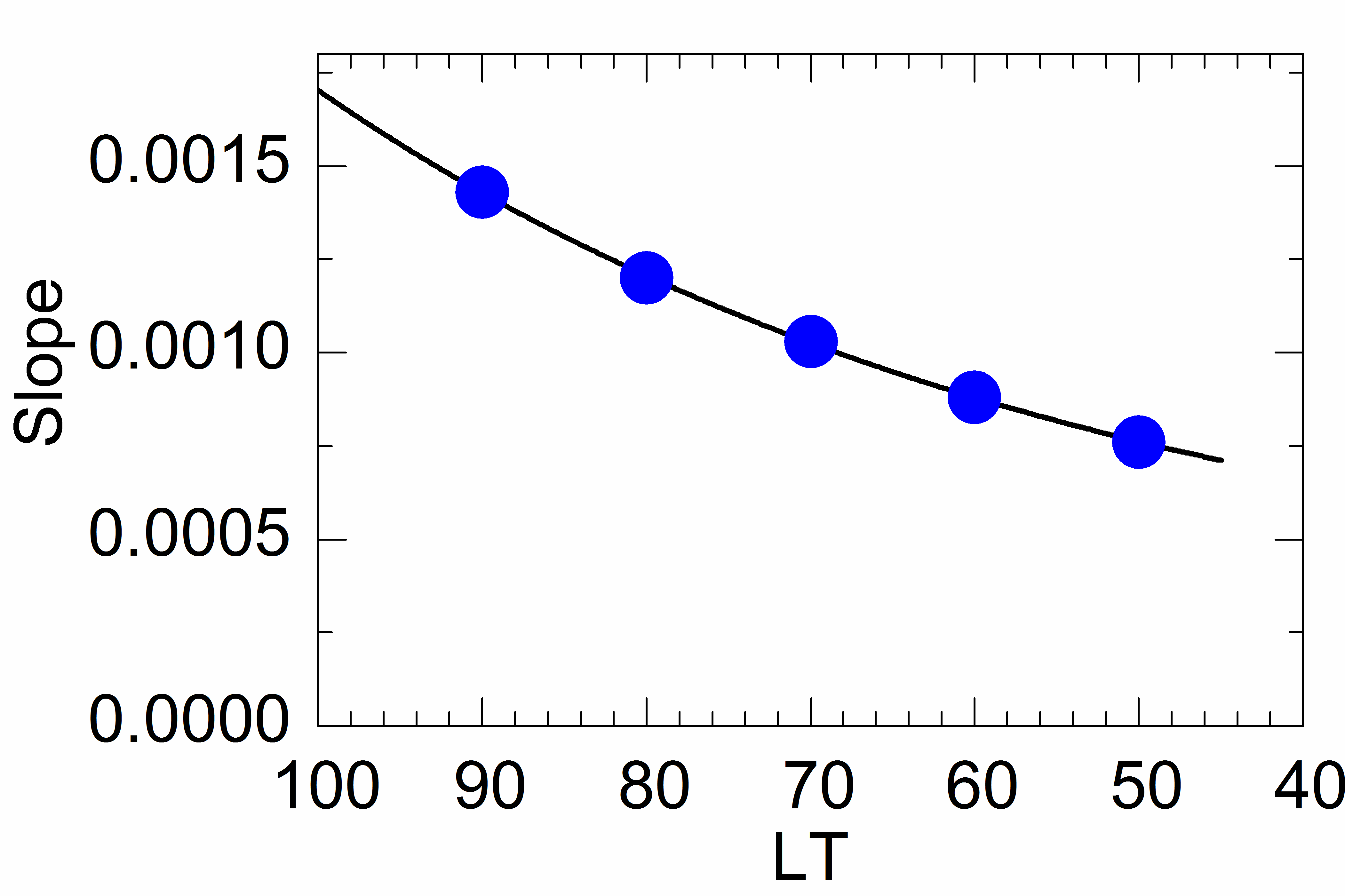


**Supplementary Figure S7 | Strength of dependence for different lifetime thresholds.** Dependence of the slopes in Supplementary Fig. S6 on LT.

**Supplementary Note 1**

The ITO substrates used for devices fabrication and contact angle measurements were all cleaned in the same manner before each series of experiments. After UV treatment for 15 min, the ITO substrates were ultrasonicated once in a bath of Semicoclean 56 (Furuuchi Chemical) for 15 min, five times in fresh bathes of purified water for 5 min each, once in a bath of acetone for 10 min, and once in a bath of isopropanol for 5 min. The isopropanol bath was boiled on a hot plate set to 240 °C before removing the substrates. Finally, the substrates were store in an oven at 85 °C until use.

**Supplementary Note 2**

We surveyed the impurities that deposit on clean Si wafers kept for different durations in the vacuum chamber of a different evaporator than the one used in the article. The evaporation sources were kept at room temperature while the Si wafers were stored in the chamber. On the surface of a wafer stored in the chamber for 30 min, 7 materials were detected using LC-MS. Storing a Si wafer in the chamber for 15 h resulted in the detection of an additional 40 materials, for a total of 47 detected materials. Thus, the substrate can be contaminated just by being in the chamber, and the amount of these impurities mixed in the device will increase with the device fabrication time. We can tentatively assign some of the signals to α-NPD, Tris-PCz, TPBi, T2T, TCTA, bis(2-ethylhexyl) adipate, and 4CzIPN. Therefore, a variety of materials, including previously deposited materials and plasticizers from the vacuum chamber components, are detected even in a vacuum chamber with different conditions when the chamber and sources are at room temperature.

**Supplementary Note 3**

The amount of adsorption was can be put into perspective with a rough estimation. First, 1 ng/cm^2^ of DOA, which has molecular mass of 370, corresponds to 1.6 × 10^12^ molecular/cm^2^. Here, we consider ITO surface as the adsorption site. The adsorption sites on the ITO surface can be estimated from the bulk density 7.12 g/cm^3^ to be 6.4 × 10^14^ molecular/cm^2^. Therefore, 1 ng/cm^2^ of DOA can covers approximately 0.3% of the ITO surface considering that the density of a thin film is lower than the bulk. About 80 kinds of the materials other than DOA were detected by LC-MS. Therefore, if the simple assumption that the total quantity of adsorbed impurities is 80 times that of DOA, impurities are estimated to cover approximately 20% of the ITO surface. This rough calculation suggests that impurities could cover several tens of percent of the ITO surface but would form less than one molecular layer. This result agrees with the measured contact angles of up to 40° since the contact angle would be expected to be higher when completely covered with impurities (the contact angle of a substrate covered with 4,4’-bis(N-carbazolyl)biphenyl is 96° [24]). Thus, the measured and calculated amounts seem reasonable, and such extremely small amounts of impurities have a large impact on the lifetime of OLEDs.

**Supplementary Note 4**

Supplementary Fig. S6 shows the dependence of normalized lifetime as a function of device fabrication time for the **Group II** devices for different final luminances relative to the initial luminance. All lifetimes were measured with a constant driving current of 10 mA/cm^2^. Supplementary Fig. S7 shows the dependence of the slopes of the plots in Supplementary Fig. S6 on LT. The slopes decrease for LT corresponding to lower final luminances, which indicates that the effect of device fabrication time is reduced for a lower final luminance. This result suggests that the initial device degradation was more strongly impacted by the extrinsic degradation arising from the impurities.
